# Missed opportunities in methanol poisoning: a qualitative exploration of the socio-material practices of health professionals responding to acute methanol poisoning in Bangladesh

**DOI:** 10.1136/bmjopen-2025-114864

**Published:** 2026-04-24

**Authors:** Janet E Perkins, Jane Brandt Sørensen, Md Shafiqul Islam, Knut Erik Hovda, Fazle Rabbi Chowdhury, Aniruddha Ghose, Abu Shahin Mohammed Mahbubur Rahman, Michael Eddleston, Alice Street

**Affiliations:** 1 Department of Social Anthropology, School of Social and Political Science, University of Edinburgh Chrystal Macmillan, Edinburgh, UK; 2 Global Health Section, Department of Public Health, University of Copenhagen, Copenhagen, Denmark; 3 Toxicology Society of Bangladesh, Chattogram, Bangladesh; 4 Department of Acute Medicine, Oslo University Hospital, Oslo, Norway; 5 University of Oslo Institute for Clinical Medicine, Oslo, Norway; 6 National Poisons Information Centre, National Institutes of Health, Oslo, Norway; 7 Department of Internal Medicine, Bangladesh Medical University, Dhaka, Bangladesh; 8 Institute for Neuroscience and Cardiovascular Research, University of Edinburgh, Edinburgh, UK; 9 Department of Medicine, Chittagong Medical College Hospital, Chattogram, Bangladesh; 10 Department of Medicine, Rajshahi Medical College, Rajshahi, Bangladesh; 11 Centre for Pesticide Suicide Prevention, and Pharmacology, Toxicology and Therapeutics, The University of Edinburgh Centre for Cardiovascular Science, Edinburgh, UK

**Keywords:** Methanol poisoning, alcohol, health service delivery, social science, Bangladesh

## Abstract

**Abstract:**

**Objective:**

This study applied a socio-material practice lens to examine health professionals’ responses to methanol poisoning in Bangladesh and to compare these practices with established guidelines.

**Design:**

This study employed a rapid ethnographic design.

**Setting:**

Data were generated in primary-level, secondary-level and tertiary-level health facilities in six districts of western Bangladesh between September 2024 and May 2025.

**Participants:**

We carried out semi-structured interviews with 50 health professionals with responsibilities for managing patients experiencing alcohol-related or poisoning-related conditions.

**Results:**

Among health professionals, the meanings of methanol poisoning as a diagnostic category, its symptoms and treatments are obscured by moral concerns about alcohol. Materials, including antidotes, for managing methanol poisoning were scarce, and health professionals reported using readily available medical supplies for supportive treatment, though not specifically adapted for methanol poisoning. Health professionals’ care practices for responding to methanol poisoning were often structured by these meanings and materials, with guidelines remaining largely invisible.

**Conclusions:**

Socio-material practices of health professionals in response to methanol poisoning in Bangladesh are characterised by missed opportunities. Improving responses requires shifting the meanings of methanol poisoning as a diagnostic category, ensuring that materials such as treatment guidelines and appropriate antidotes, such as ethanol and fomepizole, are available and supporting providers to enact care practices that reflect these guidelines.

**STRENGTHS AND LIMITATIONS OF THIS STUDY**

- This study employed a rapid ethnographic design, including semi-structured interviews with health professionals to generate an in-depth understanding of responses to methanol poisoning.
- We applied a socio-material practice lens to understand health professionals’ responses to methanol poisoning as routinised enactments that depend on the interconnection of meanings, materials and practices.
- Due to the unavailability of diagnostics, we were not always able to confirm methanol poisoning as a diagnosis.
- Health professionals in the tertiary-level hospital may have had more familiarity with methanol poisoning as a trial to test a novel diagnostic device for the conditions was being set up at the time of interviews.

## Introduction

 In November 2024, acute methanol poisoning captured international attention when it claimed the lives of six young tourists travelling to Laos from the UK, Denmark and Australia.^1^ Methanol poisoning occurs primarily when methanol, a synthetic chemical structurally similar to ethanol, is consumed inadvertently through contaminated alcoholic beverages—in this case, liquor provided for free at a hostel. When ingested, alcohol dehydrogenase, the enzyme that catalyses the breakdown of alcohols, converts methanol into the toxic metabolite formate, which accumulates and causes metabolic acidosis and cell disruption.^2 3^ Without prompt medical attention, methanol poisoning results in permanent disability and death, with reported case-fatality exceeding 30%.^4^ As little as 30 mL—two tablespoons—is potentially lethal, and lower quantities can result in irreversible blindness and brain damage.^5^,^8^

Methanol poisoning tends only to garner international attention when it affects tourists from high-income countries or when it reaches sufficient scale, which likely obscures its scope and pervasiveness. No reliable epidemiological data exist to illuminate its global burden; however, there is reason to believe the cases that come to light are the tip of an iceberg.^9 10^ Existing evidence suggests that methanol poisoning is disproportionately represented in low- and middle-income countries (LMICs) where health systems are least prepared to respond,^11 12^ most often affects men, and is rising.^9 13^

As in other LMICs, data on methanol poisoning are not routinely gathered in Bangladesh. Understanding methanol poisoning in the country is further complicated by the fact that alcohol is almost entirely prohibited, both legally and socially. However, several studies have reported on its clinical management, and reports of outbreaks consistent with the characteristics of methanol poisoning punctuate national and local news media, suggesting it is a considerable problem.^14 15^

Most deaths and disability resulting from methanol poisoning can be prevented through well-established, timely medical responses. Médecins Sans Frontières has published guidelines for responding to methanol poisoning for international application, and national guidelines for its management in health facilities in Bangladesh were issued in 2019.^16 17^ These guidelines recommend initiating treatment as soon as methanol poisoning is suspected, based on a history of alcohol consumption and presentation with one of the characteristic symptoms: hyperventilation, visual disturbance or a severe hangover in the day(s) following drinking which worsens rather than improves. The gold-standard treatment is fomepizole,^18^,^20^ a drug added to the WHO’s Model List of Essential Medicines in 2013.^21^ However, global production and limited demand mean that this medicine is expensive and rarely available in LMICs. Where fomepizole is unavailable, pure ethanol is recommended as an alternative effective treatment.^22 23^ Folic or folinic acid, bicarbonate and supportive treatment measures are also recommended, as is haemodialysis, if available.^16 17 22 24^

Health professionals play a crucial role in the effective implementation of diagnostic and treatment guidelines.^25 26^ Little is known regarding how health professionals in Bangladesh put methanol poisoning guidelines into practice. This study followed a rapid ethnographic design to generate an in-depth understanding of health professionals’ responses to methanol poisoning. Following social scientists and public health scholars who call for public health approaches to behavioural change to move beyond individualistic conceptualisations,^27^,^29^ we employ a socio-material practice lens to understand health professionals’ responses to methanol poisoning. Rather than rooted in individual behaviours, such an approach captures the ways health service providers deliver care as routinised enactments that depend on the interconnection of meanings, ie, understandings and past experiences, materials, that is, objects and infrastructures, and practices, embodied enactments of care.^27 28 30^ In this study, we examine the socio-material practices of health professionals in response to methanol poisoning in Bangladesh to understand how these compare to established guidelines. Such an understanding is crucial for shaping conditions that support health service providers in responding to methanol poisoning in line with evidence-based practice.

## Methods

### Study design

This study employed a rapid ethnographic methodological orientation. The study reporting adheres to the Consolidated Criteria for Reporting Qualitative Research (COREQ)^31^ and the criteria recommended by Gertner *et al* for reporting on ethnographic studies in implementation science.^32^

This study was embedded within a larger clinical trial evaluating a novel Formate Point-of-Care Test (Formate POCT) designed to streamline the diagnosis and treatment of methanol poisoning. Using a single drop of blood, this device detects the presence and level of formate, methanol’s toxic metabolite, in the bloodstream.^33 34^ The ethnographic sub-study we report on here was initiated before the commencement of the randomised control trial to understand the social, cultural and health systems context of methanol poisoning in Bangladesh. We carried out data generation from September 2024 to May 2025 among health professionals, law enforcement officials, representatives of groups typically considered as alcohol consumers, family members of those affected by methanol poisoning and methanol poisoning survivors. This article presents data generated among health professionals.

### Study site

This study was conducted in two locations in Bangladesh. Rajshahi division, comprising eight districts, was selected as it is the location of Rajshahi Medical College Hospital (RMCH). This 1200-bed hospital boasts one of the best-equipped haemodialysis units in the country and was selected as the first site for introducing the Formate POCT through the clinical trial. In addition to RMCH, data were generated in nearby district hospitals and *upazila* (sub-district) health complexes (UHCs). We also interviewed health managers and providers from private health institutions.

Data were also generated in Kushtia, a district adjacent to Rajshahi. It was selected based on a 2023 outbreak of what appeared to be methanol poisoning reported in national news outlets. Data were collected at the 250-bed district hospital and the five UHCs. Additionally, researchers interviewed health managers and providers from private health clinics and hospitals within the district.

### Study population

We used purposive and snowball sampling to identify interview participants for this study. We selected participants with responsibilities for managing patients experiencing alcohol-related or poisoning-related conditions. In total, we interviewed 50 health professionals for this study. The sample size was determined by data saturation: we continued to interview health professionals across categories until no new themes or insights emerged from subsequent interviews. We sampled across a range of health professionals to account for variation in terms of work experience, type of medical staff, that is, public health managers responsible for managing health facilities (n=19), medical doctors and specialists (n=9), auxiliary health service providers such as nurses (n=13) and a sub-assistant community medical officer with training equivalent to a paramedic (n=1), private health service providers and managers (n=9), and work location, that is, medical college hospital, district hospital and sub-district hospital and private health sector facilities (see table 1). None of the potential participants we approached refused to participate.

**Table 1:** Research participants

Research participants	Number
*Health managers*	
Medical college hospital managers	6
District hospital managers	2
District health managers (Directorate General of Health Services (DGHS) civil surgeons)	3
Sub-district hospital managers	8
Private health facility managers	3
*Health service providers*	
Doctors—medical college hospital	5
Doctors—district hospitals	1
Doctors—sub-district hospitals	3
Nurses—medical college hospital	6
Nurses—district hospitals	1
Nurses—sub-district hospitals	6
Sub-assistant community medical officers (SACMO)	1
Private health service providers	5
Total	50

### Procedures

Data were generated using semi-structured interviews (see online supplemental files 1; 2) and lasted approximately 45 min. Interviews explored health professionals’ perceptions of alcohol, alcohol-related poisoning, experiences managing patients presenting with alcohol-related conditions, their understanding of and experiences with methanol poisoning and managing patients presenting with suspected methanol poisoning. Interviews were conducted in a private location within the participants’ workplaces. After obtaining consent, we audio-recorded interviews. We did not carry out repeat interviews with participants. We returned interview transcripts to participants for review only on request.

All data were generated by two trained anthropologists, a postdoctoral research fellow from the UK (JEP—female) and a research associate from Bangladesh (MSI—male), who had extensive experience conducting social science research and hospital ethnography. They remained attentive to how conducting data generation as non-health professionals might influence data collection among health professionals. To address any challenges, they maintained communication with on-site clinical team members and relied on them for access and to bolster legitimacy. They also maintained close contact with the international interdisciplinary research team, comprising social scientists, toxicologists, clinicians and public health practitioners throughout data generation. All interviews were carried out in Bangla, the local language.

### Data management and analysis

We followed an inductive thematic approach for data analysis.^35^ Recorded interviews were transcribed verbatim into Bangla, cross-checked for accuracy, de-identified and translated into English. Preliminary data were analysed iteratively during data generation. This allowed the research team to refine data generation, explore unanticipated and emerging concepts and assess data saturation.

After data saturation was achieved, data analysis was led by two authors (JEP and MSI). Raw data were analysed first by JEP and MSI. We started by reading the transcripts several times to gain an understanding of the overall meanings conveyed. After this initial familiarisation with the data, we used Excel to extract and organise the data by meaning, materials and care practices, and began coding sub-themes within each category. The first two transcripts per category were coded by two authors (JEP and MSI), and a consensus was reached on coding through discussion. Thereafter, transcripts were coded by one researcher. Discussions of emerging sub-themes and data categorisation were held within the wider research team, and interpretations were formulated and validated through ongoing team meetings. Interview data were integrated with fieldnotes and observational data to contextualise, nuance and enrich them. Emerging themes were explored through reflexive practice and consultations with the wider research team. This meant that a broad range of perspectives were brought to data interpretation. Team members remained aware of their own academic positionalities and worked through different ways of seeing the data. This interdisciplinarity was a strength in the research process and enhanced the dependability, confirmability and trustworthiness of the data.

### Patient and public involvement

We involved the public and patients in the study from its outset by holding a national workshop to design the study with health policymakers and programme managers, including a patient. This allowed for their concerns to be integrated into the study design. During interviews with patients, we systematically asked them about their reflections on the study and what types of information they felt would be important, and we integrated these concerns iteratively into the research instruments.

### Role of the funding source

The funder of this study had no role in the study design, data generation, data analysis, interpretation or writing of the report.

## Results

Our findings indicate that, in Bangladesh, the socio-material practices among health professionals in response to methanol poisoning result in critical missed opportunities to save lives and restore health among those affected by acute poisoning. Select representative quotes and their conceptual relevance are tabulated (online supplemental file 1).

### Meanings of alcohol and methanol poisoning

We found that social meanings of alcohol shaped medical categorisations of methanol poisoning, and that health professionals rarely differentiated between methanol and ethanol. Among respondents, methanol poisoning was commonly conflated with conditions related to alcohol consumption, which most managers and providers had some previous experience managing. In many cases, health professionals did not recognise methanol poisoning as a distinct diagnostic category. Instead, medical incidents resulting from alcohol consumption were generally assumed to be alcohol poisoning. While this was often attributed to excessive alcohol consumption, in some cases, participants mentioned that the alcohol consumed may have expired (although alcohol does not expire) or that something harmful may have been mixed into it.

Health professionals at the most specialised end of clinical treatment pathways, such as RMCH-based nephrologists and eye specialists, were those most likely to describe methanol poisoning as a distinct diagnostic category. Medical officers and health managers, in contrast, demonstrated variable familiarity with methanol poisoning, while no auxiliary health staff recognised methanol poisoning as a diagnostic category. Medical doctors who identified methanol poisoning as a diagnostic category mentioned having heard of this during the toxicology component of their pre-service training.

Most health service providers reported that many of their previous experiences with alcohol-related conditions coincided with the religious festivals of Eid, celebrated by the country’s Muslim majority and Puja, celebrated by the significant Hindu community. They describe these as moments when they are particularly alert to the possibility of patients presenting with conditions attributable to alcohol consumption.

Moral associations with alcohol shaped the perceived appropriateness of potential treatments. Few health professionals were familiar with the use of ethanol as a clinical treatment. When presented with this proposition, many health managers and providers expressed hesitancy towards the hypothetical use of ethanol for clinical treatment. Several suggested that if ethanol were stored in the facility, it would be consumed for non-clinical purposes, and people would become addicted to alcohol. In many cases, providers anticipated that patients and their families would exhibit social and ethical reservations about their use of ethanol. Some health professionals mentioned that it would be sensitive and complex to introduce ethanol as a treatment, as Bangladesh is a Muslim-majority country. Moreover, a couple of health professionals mentioned that in their areas, there is an underlying level of health system distrust in the communities, which may be compounded if they were to use ethanol and something went wrong.

In a few exceptions, medical professionals expressed willingness to adopt ethanol for medical use in the facility if it were clinically recommended and included in national guidelines. However, they were not familiar with this national recommendation. Non-physicians also reported that they would have no objection to using ethanol if prescribed by a physician, as they are accustomed to following any treatment prescribed by doctors.

### Materials for managing methanol poisoning

Materials available for health professionals to respond to methanol poisoning in accordance with national guidelines were sparse. None of the health facilities had a copy of the national guidelines or their associated materials, such as the poster presenting the recommended algorithm for identifying and responding to suspected methanol poisoning. The guidelines were also not accessible through web searches at the time of research.

According to research participants, RMCH was the only facility, public or private, that offered tests that support the identification of methanol poisoning in the region, specifically arterial blood gas (ABG) tests. This test measures blood oxygen level, carbon dioxide in the blood, and blood acid-base status (including pH levels). If blood pH levels are too low, this may signal a metabolic acidosis (acidic blood from non-respiratory causes) due to methanol toxicity. These results are used to guide the decision-making for haemodialysis (another treatment for methanol poisoning). Despite the urgent nature of methanol poisoning, according to our respondents, ABG tests in RMCH had some practical limitations, as patients relied on the limited presence of physicians to order the tests and laboratory technicians to run them.

At the time of data collection, fomepizole was not available in Bangladesh. Thus, available recommended treatment options included folic or folinic acid, bicarbonate, ethanol and haemodialysis. In the public health sector, haemodialysis is only provided at RMCH. Here, patients whose ABG test results suggested metabolic acidosis were referred to the nephrology department for haemodialysis.

While several physicians working at RMCH and one in a district hospital mentioned ethanol as a potential treatment for methanol poisoning, ethanol was not available in any of the health facilities. If doctors prescribed ethanol, which occurred on a couple of occasions in RMCH, they instructed the patient’s attendants to obtain liquor from the local tourist bar. Despite having prescribed ethanol, no health professional shared concrete accounts of having successfully administered it.

Instead, health professionals used medical materials readily available for supportive treatment. They tended to mention ‘normal treatment’ as the standard for all alcohol-related conditions. This was often described as administering saline and gastric medication injections, which are commonly available in health facilities, though not specifically recommended for treatment of methanol poisoning. Health professionals also, though rarely, mentioned using atropine for treatment, which is reliably available in health facilities for treating organophosphorus compound (OPC) poisoning, but irrelevant for methanol poisoning. In a couple of cases, gastric lavage was also mentioned as a treatment, though also not recommended for methanol poisoning.

Nearby medical colleges hospitals, such as Pabna Medical College Hospital and Kushtia Medical College Hospital, did not provide haemodialysis services and referred patients to RMCH. Providers and managers in private health sector facilities unanimously reported that they did not have any materials for managing patients with serious alcohol-related conditions. They described referring such patients to the nearest district hospital or medical college hospital. Thus, materials for responding to methanol poisoning were highly concentrated not only in the public health sector, but in a single tertiary-level care facility in the public health sector.

### Care practices for responding to methanol poisoning

The national guidelines were largely invisible among health professionals. Only one respondent, a medical officer at RMCH, had heard of the national guideline on managing methanol poisoning, but was not familiar with its contents. Thus, care pathways were structured along other lines.

Participants reported that diagnosis of alcohol-related conditions was achieved primarily through history taking and clinical diagnosis. Health professionals were alerted to the potential involvement of alcohol in an acute health condition if the patient or family mentioned recent alcohol consumption, particularly around religious festivals. Yet many health professionals perceived that patients and families were reticent to divulge this information due to the legal and social restrictions on alcohol consumption.

Clinical assessment most commonly resulted in a distinct methanol poisoning diagnosis, rather than alcohol poisoning, in RMCH, where health professionals were most likely to conceive of methanol poisoning as a distinct diagnosis with specific clinical features. In a couple of cases in RMCH, a combination of identifying visual disturbances and the history of alcohol consumption, without the use of diagnostic tests, led health service providers to diagnose methanol poisoning and document this on patient records.

Outside RMCH, the few physicians and health managers who were familiar with methanol poisoning tended to mention its symptoms as those not highly characteristic of it. This included foaming at the mouth, more commonly associated with OPC poisoning, and stomach perforation, more commonly associated with chronic alcohol abuse or intake of corrosive agents. They rarely mentioned hyperventilation and visual disturbances. In one case, a resident medical officer posted to a UHC reported examining a patient with a history of alcohol consumption who complained of visual disturbances. He sent the patient home, advising him rest. Later, we learnt from doctors at RMCH that when the patient’s condition did not improve, his daughter took him to a private diagnostic centre in Rajshahi. When the private physician learnt the man had consumed alcohol and had visual disturbances, he immediately referred him to RMCH. There, the patient underwent haemodialysis. Although he survived, his vision was only partially restored. Lab data obtained through the project later confirmed that he was suffering from methanol poisoning.

Thus, health professionals’ care practices for responding to methanol poisoning were often structured by meanings of alcohol and other types of poisoning, and hence understandings of standard treatments for alcohol overdose and toxicity, and the use of readily available materials. Methanol poisoning guidelines were generally not considered in guiding care practices.

## Discussion

Using a lens of socio-material practices allows us to understand health professionals’ responses as routinised patterns of daily life, which are ‘carried’ by those who enact them.^27 29 30^ The findings of our study suggest that the socio-material practices structuring responses to methanol poisoning among health professionals in Bangladesh result in numerous missed opportunities to effectively address the health condition, notably by not fully using treatment guidelines and integrating ethanol therapy into treatment workflows.

National and international guidelines can serve as tools for shifting care practices in healthcare settings.^36^ Bangladesh is exemplary as one of the few countries with national guidelines for responding to methanol poisoning, despite a glaring paucity of international guidance.^17 37^ These guidelines were produced during the COVID-19 pandemic in response to a wave of methanol poisoning outbreaks in the capital. As in other settings, the consequences of COVID-19 on disrupting supply chains coincided with increased alcohol consumption and, ultimately, increased consumption of alcohol products containing methanol.^38^,^41^ These outbreaks heightened the political will to develop guidelines, which were successfully translated into action to generate and endorse them.

However, our study indicates that the guidelines have not yet percolated into the socio-material practices of health professionals, as only one of our respondents was aware of their existence. As others have documented, the existence of guidelines is not sufficient for reshaping care.^42^ Such guidance must be tailored to resource-constrained settings and sustained effort is required to support implementation in health facilities.^43^ This may be particularly challenging for health issues such as methanol poisoning, which are often invisible among health professionals, as demonstrated by our study. This has potentially been further complicated in Bangladesh, as the country has experienced recent, rapid political transformations, resulting in a turnover of health officials. Rather than viewing this as a failure, as the guidelines themselves are to be celebrated, it is a reminder to Ministries of Health that guideline development should be accompanied by a clear, well-resourced plan to ensure that they are implemented where intended. This is optimised when guidelines include training modules and an actionable, resourced training plan. These plans should include hospital administrators and prioritise their buy-in and support.

Our study found the meanings of methanol poisoning as a diagnostic category, its symptoms, and treatments, to be entangled with the meanings of alcohol. While this study does not allow comparison with other settings, clinical studies examining methanol poisoning in other LMICs suggest that it is not routinely identified and treated.^11 12^ Furthermore, the way methanol poisoning is under-addressed in global health policy and programming suggests that this is unlikely to be much different in other settings.^44^ Other studies have found alcohol-related conditions to be challenging to address in Muslim-majority societies, given the unfavourable social valuations of alcohol.^45^

While expanding the coverage of fomepizole and haemodialysis are long-term strategies for improving responses to methanol poisoning, ethanol is an already present material that can be better leveraged for treatment. However, moral associations of alcohol contributed to a reticence towards using ethanol as a treatment and its integration into care practices. Our study suggests that it will be crucial to collaborate with health managers and physicians to address their concerns regarding the availability and therapeutic use of ethanol. In contrast to many countries with strict legal prohibitions of alcohol, Bangladesh has a nationalised alcohol distillery serving a domestic market.^46^ This could be leveraged to produce ethanol for clinical use. Changing the materiality of the product, including the packaging, could alleviate moral hesitance.

Because methanol poisoning symptoms can resemble other conditions, it is essential to cultivate what Al aseri and Altamimi refer to as a ‘high index of suspicion’ of methanol poisoning among healthcare providers.^47^ This can be achieved by interventions that shift the meanings of methanol poisoning among health professionals as a category signalled by specific symptoms.^16 17^ Raising the profile of methanol poisoning through pre-service and in-service training may be crucial to this effort. Health professionals were enthusiastic about the possibility of having diagnostic materials, such as a POCT.

While current guidelines suggest initiating treatment based on suspicion, diagnostic devices, such as the Formate POCT, could be pivotal for materially confirming diagnosis and justifying treatment with ethanol.^16 17^ Diagnostic devices have risen on the global health agenda in recent years,^48 49^ reflecting increased attention to diagnosis, which was long undervalued in global health policy and practice.^50^,^52^ Made even more popular during the COVID-19 pandemic,^53^ point-of-care devices have been increasingly proposed as a solution to circumvent often slow and fragmented lab-based diagnostic services in LMICs, accelerate treatment, and improve healthcare overall.^54 55^ Despite the enthusiasm, social science scholars have demonstrated that these diagnostic devices often operate differently from what their designers expect, and reshape care in unintended ways.^56 57^ Nonetheless, they hold considerable potential to expand access to healthcare and promote health equity. If the Formate POCT trial proves successful, this device may be crucial in rendering methanol poisoning visible, and ‘uncollapsing’ the meanings of methanol poisoning. This may also contribute to shifting moral orientations to justify the use of ethanol as a life-saving treatment. Furthermore, the Formate POCT is a small device that can be easily mobilised at different levels of the health system, with the potential to initiate life-saving and disability-averting treatment even before patients reach tertiary-level health facilities.

As a social science qualitative study, our objective was not to achieve generalisability in our findings, but instead to provide an in-depth exploration of methanol poisoning responses in our field site. Nevertheless, this study’s findings have transferable value across LMIC contexts, and Muslim-majority settings in particular. They can alert health actors in these settings to the social-material practices that may be influencing health service responses to methanol poisoning, for example, to the ways moral orientations towards alcohol might shape both meanings of methanol poisoning and the use of ethanol as treatment. They also underscore the need for increased attention to move alcohol-related treatment guidelines from the policy level into the implementation level in Muslim-majority settings.

A significant strength of our study is that, to our knowledge, it is the first to provide a qualitative examination of methanol poisoning in LMICs and of health professionals’ responses to it. Further qualitative research is urgently needed to understand variations in health professionals’ responses to methanol poisoning in different LMIC contexts, critical for designing context-specific actions to improve these responses. This type of research is equally needed to bring attention to methanol poisoning as the endemic scourge that is claiming the lives and health of the most vulnerable, rather than an exceptional condition that primarily affects tourists from HICs, as news media representations typically portray. This will also support its integration into global health policy and programming agendas, where it has largely been invisible to date.

A key limitation of our study is that, in most alcohol-related cases, we could not confirm whether methanol or another factor was responsible. Methanol poisoning test results were confirmed in only several cases by the project team, as this study was conducted prior to the implementation of the Formate POCT trial. Embedding qualitative research in future clinical studies capable of confirming methanol poisoning can provide further understanding of the specificities of methanol poisoning. Another limitation is that this study was conducted in the context of a methanol-poisoning research trial. Thus, some participants may have been more exposed to information on methanol poisoning than they were prior to the trial setup. However, this seems to be a minimal factor, as few health service professionals were familiar with methanol poisoning.

## Conclusion

Despite its common representation as a ‘tourist’ affliction, limited available evidence suggests methanol poisoning most commonly occurs in LMICs and appears to be on the rise. It is therefore critical that health systems be prepared to respond to this type of poisoning in these settings. Our study found that the socio-material of practices of health service providers in Bangladesh widely result in missed opportunities to save lives and restore the health of those affected. The meanings of methanol poisoning as a diagnostic category, its symptoms and treatments are obscured by the meanings and moral concerns about alcohol. Most health professionals conflate methanol poisoning with alcohol poisoning, rather than understanding it as a distinct diagnosis with a separate treatment pathway. Moreover, while pure ethanol is a potent antidote for methanol poisoning, few health professionals were familiar with this therapeutic use, and some expressed hesitancy to stock and use ethanol for clinical treatment on moral grounds. Materials for managing methanol poisoning, even those locally available, are scarce in health facilities. These include national guidelines and algorithms published in 2019 that have not reached health facilities, as well as antidotes, including ethanol, which is locally available but not stored or used in health facilities. Health professionals’ care practices for responding to methanol poisoning were structured by these meanings and materials, with guidelines remaining invisible in shaping care.

While improving health system responses to methanol poisoning in LMICs may not be easy, as far as global health issues go, its solutions are relatively simple. They do not require new clinical knowledge, scientific innovation, or highly complex treatments. Instead, they call for a reshaping of socio-material practices that involve understanding methanol poisoning as a diagnostic category, ensuring that materials are available, and reshaping care practices to respond to methanol poisoning. This can be done without attempting society-wide understandings of alcohol or fundamentally reconfiguring health systems. Addressing these missed opportunities can make the difference between life and death, or between a life with or without serious disabilities, for innumerable people throughout the globe. It is high time to cultivate social practices that seize these opportunities to save lives.

## Supplementary material

10.1136/bmjopen-2025-114864 online supplemental file 1

10.1136/bmjopen-2025-114864 online supplemental file 2

## Data Availability

Data are available upon reasonable request.
