# Supplementary material for: Missed opportunities in methanol poisoning: a qualitative exploration of the socio-material practices of health professionals responding to acute methanol poisoning in Bangladesh
Source: BMJ Open. 2026 Apr 24;16(4):e114864. doi: 10.1136/bmjopen-2025-114864 (PMC13110524; doi:10.1136/bmjopen-2025-114864)
Supplement: online supplemental file 1 [file bmjopen-16-4-s001.docx]

# Supplementary File 1: Illustrative table of quotes

| **Conceptual relevance** | **Quote** | **Research participant** |
| --- | --- | --- |
| **Meaning: social significance and previous experience** | | |
| Conflation of methanol and ethanol poisoning | Respondent: Yes, I have heard of methanol poisoning. Interviewer: What exactly is methanol poisoning?  Respondent: Methanol poisoning, you see, it's like another type of poison. But from what I understand, it seems to be a bit less potent than other poisons.  Interviewer: Alright, okay. So, do you know the difference between ethanol and methanol?  Respondent: Ethanol?  Interviewer: Yes. I mean, what's the difference between regular alcohol and poisonous alcohol?  Respondent: I'm not quite grasping what you mean by regular alcohol and poisonous alcohol. Could you clarify what you consider poisonous alcohol and what you consider regular alcohol? ... When we say poisonous liquor, well, I don't really know it in that way, or what the names are, or what it's actually like. So, everyone who comes in says they've been drinking liquor. So I have seen patients who have consumed alcohol. But I myself don't know if there are any differences between types of alcohol. | Nurse, RMCH |
| Conflation of methanol and ethanol poisoning | Respondent: Alcohol poisoning cases are quite infrequent. For instance, we don't see any cases before Eid. However, in the week leading up to Eid, we do get some, though none so far this week. Alcohol-related issues are generally less common, probably because people consume it in limited quantities, Interviewer: Have you heard about methanol or methanol poisoning? Respondent: I haven't heard of methanol poisoning, no. Interviewer: Have you ever heard about methanol poisoning, perhaps in the context of alcohol poisoning? Interviewer: No, I haven't specifically heard of methanol poisoning; I've only heard about alcohol poisoning in general. We've heard of cases where people get poisoned by alcohol or drugs, but none of us have heard of methanol poisoning. | Nurse, RMCH |
| Conflation of methanol and ethanol poisoning | Respondent: If I think back to my internship, we encountered an incident involving alcohol. The alcohol was likely poisonous for some reason. Once, as a student at Rangpur Medical College, we saw many people die in Rangpur. Approximately 28 or 29 people died at once due to that poisoning. Interviewer: Approximately around what year? Respondent: It was towards the end of 1999, or around the year 2000, something like that. After that, when we were in Rajshahi, we again saw that people died due to alcohol during various festivals in Rajshahi. In Kushtia, we had a couple of cases, no deaths. But we handled two or three cases of alcohol toxicology. Patients were admitted here with toxic effects. We have seen that here. After coming here, we have had alcohol patients, but they did not die. Many times it was due to over drinking. Some might have consumed something bad. Because of this. | Medical officer, Upazila Health Complex, Kushtia |
| Methanol poisoning as distinct diagnostic category | Respondent: Regarding your question about the prevalence of alcohol consumption, in our daily practice, we observe that alcohol or methanol poisoning accounts for about five to ten percent of all poisoning cases. That's my observation, and it's unlikely to be higher. However, in Rajshahi as a whole, within the community outside the hospital setting, alcohol consumption is not uncommon Interviewer: Is methylated spirit drinkable? Respondent: No. It's not meant for consumption. However, it produces a similar intoxicating effect to alcohol, but with far more severe side effects. Interviewer: Is there any safe amount of methylated spirit that can be consumed? What quantity becomes toxic? Respondent: Methylated spirit is not safe for consumption in any quantity. It's not meant for drinking, regardless of the amount. Interviewer: What amount could be fatal? Respondent: Around a hundred... isn't it a hundred millilitres? That's what I've always known. As far as I know, a hundred millilitres of methylated spirit can be fatal. Even 14 ml can be lethal...Sadly, many people are unaware of the dangers of methylated spirits. They don't realize that it's often banned by the government and that it can cause severe harm. Due to this lack of awareness, they consume it without understanding the consequences. | Medical Officer, RMCH |
| Negative moral valuation of ethanol as clinical treatment | There are some—there's some religious sensitivity around [using ethanol as a medical treatment]. There's a cultural barrier to using ethanol as a medicine. I mean, this barrier can be overcome, but we need strong evidence that it is methanol poisoning and that we should give ethanol. | Professor of Medicine, RMCH |
| Negative moral valuation of ethanol as clinical treatment | If we give ethanol to counteract the methanol poisoning, giving alcohol here... Alcohol is forbidden in our religion...If it were just within my ward, and my nurse, the supervisor, myself, and the senior doctor knew, then we could potentially apply it to the right patient. But as soon as more people become aware of it, it will become difficult for me to use it. If people know we're administering ethanol, they'll think we're just giving them alcohol. Our mentality hasn't quite reached a point where we can easily accept it, or we're not educated enough about it. Then there's also the Islamic perspective, where people will say we're giving alcohol, which is forbidden. | Health manager, Upazila Health Complex, Kushtia |
| **Materials: Objects and Infrastructures** | | |
| Diagnostic materials | Interviewer: Are there any confirmatory lab tests to diagnose methanol poisoning? Respondent: In our current practice, we don't routinely send samples for specific methanol testing. We lack those facilities. Perhaps there's a newer test available elsewhere. Interviewer: Do you ever face ambiguity in diagnosing these cases? Respondent: Yes, there's always a degree of uncertainty. Our facilities don't allow for definitive testing. Interviewer: Despite that, you still have to provide treatment. Respondent: Yes, we do. The patient's medical history plays a crucial role. Then, we rely on their signs and symptoms. If symptoms are severe, there's limited intervention possible. The history is vital. | Medical Officer, RMCH |
| Treatment materials | Actually, I think the treatment is usually normal treatment. Especially in my experience, from what I've seen, if it's not something very specific, I'm not sure what exactly they give, but I've seen that they receive normal treatment. I particularly see them give omeprazole or gastric injections, and also saline. | Nurse, Male ward, RMCH |
| Treatment materials | Interviewer: Alright. And have you ever heard if people can die from alcohol poisoning, or if there's a risk of death? Respondent: Yes, brother, it can certainly happen with alcohol. Just like I mentioned, a fifteen-year-old child came in... The child was very restless when he arrived, so the doctor immediately told me to administer 20 doses of atropine. After giving him 20 doses, the child's condition worsened significantly. When that happened, I told the doctor that for a fifteen-year-old, maybe we should have started with 10 doses instead of 20. Because right after I administered the 20 doses, the child became very ill, and that child actually died from alcohol poisoning. He came in with alcohol poisoning, you see? | Nurse, RMCH |
| Treatment materials | Interviewer: What antidote do you use for methanol poisoning? Respondent: We administer ethanol as an antidote for methanol poisoning. Interviewer: Anything else besides ethanol? Respondent: No, we only use ethanol. Interviewer: Who typically obtains the ethanol, and how is it acquired? Respondent: I'm not entirely sure. Based on an incident my colleague mentioned, they obtained ethanol from a tourism-related source. We provided them with a requisition, along with medical documents, and they collected it from there. It seems ethanol is available in tourism-related settings. | Medical Officer, RMCH |
| Treatment materials | I believe a more significant concern than methanol poisoning itself is the development of respiratory and renal complications, such as renal impairment and acute kidney injury (AKI). These patients often require haemodialysis or hemoperfusion. While we primarily focus on immediate symptomatic management—because we often can't administer timely antidotes like fomepizole or ethanol due to availability issues—these complications necessitate specialized care. | Medical Officer, RMCH |
| Treatment materials | Respondent: Ethanol can act as an antidote for methanol poisoning, although we don't use it as such here. We primarily provide conservative care.  Interviewer: Why don't you administer ethanol as an antidote? Respondent: It's simply not available at our facility.  Interviewer: Have you ever attempted to prescribe it?  Respondent: No, I haven't. We haven't encountered patients with severe encephalopathy, a condition that might warrant its use. Patients usually present with disorientation, which improves with conservative treatment | Medical officer, District Hospital Kushtia |
| **Care practices** | | |
| Familiarity with guidelines | Interviewer: Do you have any specific guidelines here in your department? Or have you heard of any national guidelines for treating methanol poisoning?  Respondent: Guidelines specifically for methanol poisoning?  Interviewer: For treatment Respondent: No, we don't have specific, locally developed guidelines that I'm aware of. | Medical officer, District Hospital, Kushtia |
| History-based diagnosis | Actually, we don't encounter methanol poisoning in a straightforward way very often. So, we usually rely on taking a patient history to determine if it's some other type of poisoning or alcohol poisoning. The main thing is to get a detailed history, and then we try to figure out what kind of poisoning it is – whether it's OPC poisoning, a herbicide, alcohol poisoning, or marijuana poisoning. We primarily deduce this from the patient's history. We also consider the timing of when the patients come in. For example, if we see someone coming in the day after Christmas, we might suspect alcohol poisoning as being more likely. Or if we see cases during the Puja festival, particularly on the day of the Hindu community's immersion ceremony, we consider alcohol poisoning as a possibility because many people consume alcohol during that time | Health manager, Upazila Health Complex, Kushtia |
| History-based diagnosis | Actually, diagnosing methanol poisoning primarily relies on the patient's history. We don't have access to investigative facilities. It's mostly based on history and circumstantial evidence. But if we had a lab tool like that, it would be very helpful for us. And we could proceed with a specific antidote, like ethanol. If indicated, we could administer ethanol. Then we should use ethanol. Currently, we can't definitively say a patient needs ethanol because we lack certainty. | Professor of Medicine, RMCH |
| Methanol poisoning symptom identification | With methanol poisoning, our primary concern is vision. We assess visual acuity to see if it's impaired, checking for pre-existing conditions or new visual disturbances. General symptoms of poisoning, such as nausea and vomiting, are common. However, visual impairment is a key indicator of methanol poisoning. | Medical Officer, RMCH |
| Identification of non-methanol poisoning-related symptoms | Respondent: He's a type of alcoholic. You're not supposed to die just from drinking regular alcohol. It's when they consume methanol spirit that their stomach, our stomach, gets perforated. Yes, if there's a large perforation, we don't have the capacity to repair it here. We refer them to Kushtia. By the time they get to Kushtia for the repair, the patient often dies. Interviewer: And sir, could there be any other symptoms, as far as you know? Respondent: With methyl alcohol poisoning, basically, there's a bit of a foam, a little foaming at the mouth. I mean foam. Foam appears, and they also become a bit... what's it called... they don't really have the typical drunken state you get with ethanol. You don't get that drunkenness with methanol. That kind of intoxication. Instead, there's more abdominal pain and unconsciousness. | Medical officer, Upazila Health Complex, Kushtia |
