# Supplementary material for: Missed opportunities in methanol poisoning: a qualitative exploration of the socio-material practices of health professionals responding to acute methanol poisoning in Bangladesh
Source: BMJ Open. 2026 Apr 24;16(4):e114864. doi: 10.1136/bmjopen-2025-114864 (PMC13110524; doi:10.1136/bmjopen-2025-114864)
Supplement: online supplemental file 2 [file bmjopen-16-4-s002.docx]

**Semi-structured interview guideline for healthcare managers and providers about their perceptions of alcohol use and poisoning resulting from “toxic alcohol”/methanol-contaminated beverages in the district**

**Introduction**

Our names are _________________ and we are from___________________. We are here to understand and learn from you about alcohol use and poisoning as resulting from “toxic alcohol” or methanol-contaminated beverages in this area. You have been chosen for this interview because you are a health service manager provider who may have encountered patients experiencing poisoning, including that which has resulted from ingesting toxic alcohol or methanol contaminated beverages. We are here to learn about alcohol use and poisoning in this area and how poisoning is managed in the health facility. We are also interested in discussing the possibility of introducing a new rapid test in health facilities to help improve the medical treatment of patients experiencing poisoning as a result of consuming toxic alcohol/methanol contaminated beverages.

Before beginning, we would like to review this participant information sheet with you and ask that you let us know if you have any questions. If you are happy to participate, we would ask you provide informed consent.

As it can be difficult to take notes fast enough, we would like to record the discussion. Then we can go home and listen to the recording and better understand and remember what you said. *[Turn on the recorder if they consent].*

| **Theme** | **Main question** | **Probes** |
| --- | --- | --- |
| Alcohol in general | What is alcohol consumption like in this area?  Who consumes alcohol in this area?  What type of alcohol do people consume in this area?  What are the positive and negative consequences of alcohol use in this area? | Is alcohol consumption common in this area? Why do you think this?  Where and when do people consume alcohol?  What religious factors influence whether people drink alcohol in this area?  What legal factors influence whether people drink alcohol in this area?  What economic factors influence whether people drink alcohol in this area?  What other factors influence whether or not people drink alcohol in this area?  Do you think alcohol consumption is becoming more or less common in this area? Why do you think this?  What types of people are thought of as more likely to drink alcohol (e.g., men/women, wealthy or working class, students, etc..)? Why these people?  What types of alcohol do people drink?  How to they access it?  Is it produced locally or nationally/internationally? By whom?  Why do they drink these alcoholic beverages?  What are the negative consequences of alcohol consumption in this area (e.g., poor health, violence, road traffic accidents)? How does alcohol play a role?  Are there any positive consequence of alcohol in this area (e.g., social bonding, relaxation)? What are they? How are these specific to alcohol? |
| Influences of alcohol on health and healthcare | How does alcohol influence the health of people in this area?  What are your experiences encountering people having consumed alcohol in the health facility?  What do health service providers tend to think about people who consume alcohol?  How do health care managers and providers treat people they suspect have consumed alcohol? | Does alcohol use lead to chronic health problems in this area? How?  Does alcohol use lead to acute health problems in this area? How?  Have you managed patients who consumed alcohol? Can you describe the experience?  Have you had experiences where you encountered family members/companions of patients under the influence of alcohol? Can you describe the experience?  What do they call people who drink alcohol?  How do they feel about managing patients or their entourage when they have consumed alcohol? Why?  Do they treat them the same as other patients? With frustration or contempt? Can you describe any experiences? |
| Poisoning in general | How is poisoning encountered and managed in the health facility (all kinds)? | How often do you encounter patients experiencing poisoning? What kinds? (e.g. snakebites, pesticides)  Can you describe one poisoning case you have encountered from beginning to end?  What groups of people are most likely to come to the hospital for treatment for poisoning (adolescents/young adults/adults/elderly, men/women, people living alone, people with a family)?  What is the source of the poison?  When is the health facility most likely to receive patients experiencing poisoning (specific time of the day, specific time of the month or year)?  How is poisoning managed in the health facility?  What types of poisoning cases are easier and more difficult to manage? |
| Explanations of poisoning resulting from toxic alcohol/methanol-contaminated beverages | Who, why and how do people experience poisoning as a result of toxic alcohol/methanol-contaminated beverages? | How often do you encounter patients experiencing poisoning resulting from toxic alcohol/methanol-contaminated beverages?  What groups of people are more likely to come to the hospital for treatment for poisoning resulting from consuming toxic alcohol/methanol contaminated beverages (adolescents/young adults/adults/elderly, men/women, people living alone, people with a family)?  How do you explain their consumption of toxic alcohol/methanol contaminated beverages?  When is the health facility most likely to receive patients experiencing poisoning resulting from the consumption of toxic alcohol/methanol-contaminated beverages (specific time of the day, specific time of the month or year)?  Where do people access toxic alcohol?  What about the alcohol makes it toxic? |
| Communicating about toxic alcohol-related poisoning | Do healthcare providers/managers discuss poisoning resulting from toxic alcohol/methanol-contaminated beverages in their work setting? | What do they discuss? (e.g., is it an important problem)  In what situations?  When and with whom (e.g., other health service providers, in health management meetings, with patients)? |
| Reaching health facilities | How do patients experiencing poisoning as a result of toxic alcohol/methanol-contaminated beverages reach the health facility? | How do people experiencing poisoning as a result of toxic alcohol/methanol-contaminated beverages typically arrive at the health facility (e.g., what is their condition, how are they transported to the health facility)?  Who brings them? (e.g., family members, community members)  What challenges do they face? How does this influence treatment in the health facility?  How is the health facility involved in overcoming these challenges? (e.g., emergency transportation) |
| Health facility responses to pesticide poisoning | How does the health facility respond when someone arrives at the health facility and is suspected of experiencing poisoning resulting from toxic alcohol/methanol-contaminated beverages?  *[Create a map to draw out the expected patient pathway]* | In your experience, where are patients suspected of experiencing poisoning resulting from toxic alcohol /methanol-contaminated beverages taken within the health facility? Who is involved in taking them to these places? How long does it take?  What symptoms alert health care providers that a patient may be experiencing poisoning as a result of consuming toxic alcohol/methanol contaminated beverages? |
| Diagnosis poisoning resulting from the consumption of toxic alcohol/methanol-contaminated beverages | How is poisoning resulting from consuming toxic alcohol/methanol-contaminated beverages diagnosed? | What symptoms/situations lead health staff to test patients for toxic alcohol consumption?  Can you describe the process for diagnosing poisoning resulting from the consumption of toxic alcohol? (use timeline/map)  What medical diagnostic tools are used in this diagnosis/confirmation?  Who is involved in making this diagnosis/confirmation?  What samples are taken for diagnosis? Who does this?  What spaces do samples travel to for diagnosis/confirmation?  What challenges are encountered in this diagnostic pathway?  What are the possible results of the diagnostic tests?  Is it possible for the results to be ambiguous or incorrect? How do health care providers become confident in the results? |
| Treatment of patients experiencing poisoning as a result of toxic alcohol/methanol-contaminated beverages | What medical treatment do patients experiencing poisoning as a result of toxic alcohol/methanol-contaminated beverages receive? | What is the protocol for treating patients experiencing poisoning as a result of toxic alcohol/methanol contaminated beverages? How does the protocol compare to what generally happens in practice?  Who is involved in the medical treatment of patients experiencing poisoning resulting from the consumption of toxic alcohol/methanol-contaminated beverages? What do they do?  What antidotes are available in the health facility to treat people experiencing poisoning resulting from toxic alcohol/methanol-contaminated beverages? (e.g. fomepizole, ethanol) Are these generally in stock? How are they procured through the health system?  What are the main challenges in providing medical treatment to patients experiencing poisoning as a result of consuming toxic alcohol/methanol-contaminated beverages? |
| Perceptions regarding those experiencing pesticide poisoning | How do health service providers perceive patients experiencing poisoning as a result of toxic alcohol/methanol-contaminated beverage consumption? | How do health service providers treat patients suffering from having consumed toxic alcohol/methanol-contaminated beverages? (e.g., with concern, contempt, stigma) |
| Expectations related to the potential introduction of the POCT | In our project, we are planning to test the introduction of a rapid test in health facilities to identify poisoning resulting from methanol-contaminated beverages more quickly  *[Show them the POCT]*  What are your thoughts on this?  How do you think this could help improve health service delivery?  What challenges/ downsides do you think occur in introducing a test like this in health care facilities? | Do you think it would be good to have such a test available in health facilities? Why or why not?  If yes, what health facilities do you think should have such a test available?  What do you think some of the advantages could be of having such a test?  How do you think it could be integrated into routine health services?  How do you think it could be used?  Do you think it would be helpful to alert other people that there is toxic alcohol being distributed in your area?  What do you think the disadvantages of having such a test would be? (e.g., legal, social)  What challenges would health facilities face in introducing a test like this? |
| Community participation | Are there any actions/activities in the area which are designed to address poisoning resulting from toxic alcohol/methanol-contaminated beverages? What are they?  How do you think health managers/providers should be involved in addressing poisoning resulting from toxic alcohol/methanol-contaminated beverages?  What do you think about our research project on this topic?  We want to make sure that what we find through this project can be beneficial in this area. What information would be helpful and important for you and other health staff to improve the situation related to toxic alcohol/methanol contaminated beverages in this area? | If yes, what are they? Who is in charge of them? What do you think about these?  If no, why not?  What types of actions/activities would you like to see in this area to address toxic alcohol poisoning?  How could health care managers/providers be involved.  Do you think it should be added to the agenda of health management meetings? Should there be standalone meetings?  Are there other ways you would like to be involved in addressing this? If yes, how? If no, why not?  Do you think it is important to gather information about alcohol use and toxic alcohol/methanol contaminated beverages in your area? Why or why not?  Do you think this research could benefit the people you serve as a health service provider/manager? How?  Do you think that this research could harm people you serve as a health service provider/manager? How?  What information would like to have on this topic?  What other people do you think we should talk to about this? |

**End the interview**

Thank you very much for your comments. We have no further questions. Do you have any questions about our research or final comments for the interview?

Thank you very much. This is very valuable information for us so thank you.

*[Turn off recorder].*
