# Supplementary material for: Missed opportunities in methanol poisoning: a qualitative exploration of the socio-material practices of health professionals responding to acute methanol poisoning in Bangladesh
Source: BMJ Open. 2026 Apr 24;16(4):e114864. doi: 10.1136/bmjopen-2025-114864 (PMC13110524; doi:10.1136/bmjopen-2025-114864)
Supplement: Supplementary file 3 [file bmjopen-2025-114864.reviewer_comments.pdf]

PEER REVIEW HISTORY

BMJ Open publishes all reviews undertaken for accepted manuscripts. Reviewers are asked to complete a checklist review form and are provided with free text boxes to elaborate on their assessment. These free text comments are reproduced below.

ARTICLE DETAILS

Title (Provisional)

Missed opportunities in methanol poisoning: A qualitative exploration of the socio-material practices of health professionals responding to acute methanol poisoning in Bangladesh

Authors

Perkins, Janet Elaine; Soerensen, Jane Brandt; Islam, Md. Shafiqul; Hovda, Knut Erik; Chowdhury, Fazle Rabbi; Ghose, Aniruddha; Mahbubur Rahman, Abu Shahin Mohammed; Eddleston, Michael; Street, Alice

---

VERSION 1 - REVIEW

**Reviewer**	**1**
**Name**	**Sandoval, Cristian**
**Affiliation**	**Universidad de La Frontera, Faculty of Medicine**
**Date**	**22-Dec-2025**
**COI**	**None**

This manuscript presents a high-quality, timely, and conceptually rigorous qualitative study examining health professionals' responses to acute methanol poisoning in Bangladesh through a socio-material practice lens. The topic addresses a critical but underexplored global health issue, and the paper is well aligned with BMJ Open's scope and standards.

Major Strengths

Original Contribution

To my knowledge, this is the first qualitative study in a low- and middle-income country examining methanol poisoning from the perspective of frontline health professionals using a socio-material framework.

The integration of anthropology, public health, and toxicology is a major strength.

Methodological Rigor

The rapid ethnographic design is well justified and appropriately reported, with adherence to COREQ and relevant ethnographic reporting standards.

Sampling is purposive, diverse, and clearly described, with convincing evidence of data saturation.

Analytical Depth

The use of meanings, materials, and care practices as analytical categories is coherent and consistently applied.

The illustrative quotes are rich, well contextualised, and clearly linked to analytic claims.

Policy and Practice Relevance

The findings have clear, actionable implications for guideline implementation, training, and resource allocation.

The discussion convincingly argues that improving responses does not require novel technologies, but rather socio-material realignment.

Ethics and Reflexivity

Ethical approvals, consent procedures, and patient/public involvement are clearly documented.

The inclusion of a reflexivity statement is commendable and aligns with best practice for global health research partnerships.

Minor Suggestions for Improvement

These points are intended to strengthen clarity and impact rather than address substantive weaknesses:

Clarify Transferability

While the discussion appropriately avoids overgeneralisation, a short paragraph explicitly addressing how findings may (or may not) transfer to other LMIC or Muslim-majority contexts would enhance interpretability for international readers.

Strengthen Visibility of National Guidelines

Consider briefly elaborating on why the 2019 national guidelines have remained inaccessible (e.g., dissemination mechanisms, digital access, training gaps), if data permit. This would further strengthen the implementation science contribution.

Terminology Consistency

Ensure consistent use of terms such as alcohol poisoning, toxic alcohol, and methanol poisoning throughout, particularly in the Results section, to avoid any residual ambiguity for non-specialist readers.

Conclusion Tightening

The conclusion is strong but could be marginally tightened by explicitly restating the three socio-material domains (meanings, materials, practices) as a final analytical takeaway.

Overall Assessment

This is a robust, well-written, and highly relevant manuscript that makes an important contribution to qualitative global health research and implementation science. The study is methodologically sound, ethically rigorous, and policy-relevant. I recommend acceptance pending minor revisions as outlined above.

Reviewer 2

**Name**	**Mostafazadeh, Babak**
**Affiliation**	**Shahid Beheshti University of Medical Sciences**
**Date**	**02-Jan-2026**
**COI**	**None**

Minor clarifications would strengthen it further, including a brief elaboration on how data saturation was determined, greater attention to researcher reflexivity, and slightly more explicit discussion of policy and implementation implications such as:

The policy implications could be made more concrete. For example:

What specific actions could Ministries of Health or hospital administrators take in the short term?

How might training curricula be modified?

The role of the ongoing POCT trial is mentioned but could be more explicitly connected to future system-level change.

**Reviewer**	**3**
**Name**	**Barták, Miroslav**
**Affiliation**	**Charles University and General University Hospital in Prague, Department of Addictology**
**Date**	**04-Feb-2026**
**COI**	**None**

Thank you for the opportunity to write this review and to the authors for covering this topic. The article certainly provides a new and important perspective on the topic of methanol poisoning, and the interviews conducted are highly informative. On the other hand, I see some fundamental problems. The first is the question of literature review. The literature on methanol poisoning is quite extensive, but the introductory section of the article fails to mention a number of publications that would be worth citing, and there is no mention of the research strategy. The design of the study is appropriate for the purpose of the article, but I believe that some of the information provided in CONSEQ is missing from the paper or is described very briefly. The methodological approach to the analysis itself is described in very general terms; the appendix contains an illustrative list of questions, but not the entire coding framework. It is not entirely clear how many people coded and what the specific analysis process was. The processing of the results should be much clearer, and visualization could make this part of the text more readable. I find the discussion insufficient, and the definition of strengths and weaknesses is inadequate. The conclusions are rather general and do not fully correspond to the possibilities of qualitative analysis, and they are very short.

With better processing of the qualitative analysis, I consider the survey to be beneficial, but a complete revision of the text is necessary.

VERSION 1 - AUTHOR RESPONSE

Reviewer: 1
Dr. Cristian Sandoval, Universidad de La Frontera
Comments to the Author:
This manuscript presents a high-quality, timely, and conceptually rigorous qualitative study examining health professionals’ responses to acute methanol poisoning in Bangladesh through a socio-material practice lens. The topic addresses a critical but underexplored global health issue, and the paper is well aligned with BMJ Open’s scope and standards.

Major Strengths
Original Contribution
To my knowledge, this is the first qualitative study in a low- and middle-income country examining methanol poisoning from the perspective of frontline health professionals using a socio-material framework.
The integration of anthropology, public health, and toxicology is a major strength.

Thank you for reviewing our manuscript and recognising its significance to global health debates.

Methodological Rigor
The rapid ethnographic design is well justified and appropriately reported, with adherence to COREQ and relevant ethnographic reporting standards.

Sampling is purposive, diverse, and clearly described, with convincing evidence of data saturation.

Analytical Depth
The use of meanings, materials, and care practices as analytical categories is coherent and consistently applied.
The illustrative quotes are rich, well contextualised, and clearly linked to analytic claims.

Policy and Practice Relevance
The findings have clear, actionable implications for guideline implementation, training, and resource allocation.
The discussion convincingly argues that improving responses does not require novel technologies, but rather socio-material realignment.

Ethics and Reflexivity
Ethical approvals, consent procedures, and patient/public involvement are clearly documented.
The inclusion of a reflexivity statement is commendable and aligns with best practice for global health research partnerships.

Minor Suggestions for Improvement
These points are intended to strengthen clarity and impact rather than address substantive weaknesses:

Clarify Transferability
While the discussion appropriately avoids overgeneralisation, a short paragraph explicitly addressing how findings may (or may not) transfer to other LMIC or Muslim-majority contexts would enhance interpretability for international readers.

- Thank you for this suggestion. We have added a paragraph on this in the discussion section.

Strengthen Visibility of National Guidelines
Consider briefly elaborating on why the 2019 national guidelines have remained inaccessible (e.g., dissemination mechanisms, digital access, training gaps), if data permit. This would further strengthen the implementation science contribution.

- Thank you for this recommendation. We have added fuller consideration of the National Guidelines, why they have not yet reached their potential, and learning for Bangladesh and beyond in the discussion section.

Terminology Consistency
Ensure consistent use of terms such as alcohol poisoning, toxic alcohol, and methanol poisoning throughout, particularly in the Results section, to avoid any residual ambiguity for non-specialist readers.

- Thank you. We have reviewed the paper's terminology for consistency and revised it accordingly.

Conclusion Tightening
The conclusion is strong but could be marginally tightened by explicitly restating the three socio-material domains (meanings, materials, practices) as a final analytical takeaway.

- Thank you for this recommendation. We have revised the conclusion accordingly.

Overall Assessment
This is a robust, well-written, and highly relevant manuscript that makes an important contribution to qualitative global health research and implementation science. The study is methodologically sound, ethically rigorous, and policy-relevant. I recommend acceptance pending minor revisions as outlined above.

Reviewer: 2
Dr. Babak Mostafazadeh, Shahid Beheshti University of Medical Sciences
Comments to the Author:
Minor clarifications would strengthen it further, including a brief elaboration on how data saturation was determined, greater attention to researcher reflexivity, and slightly more explicit discussion of policy and implementation implications such as:
The policy implications could be made more concrete. For example:
What specific actions could Ministries of Health or hospital administrators take in the short term?
How might training curricula be modified?
The role of the ongoing POCT trial is mentioned but could be more explicitly connected to future system-level change.

Thank you for reviewing our manuscript and for helpful comments. Based on this feedback, we have made the following changes:

- Data saturation: On page 6, we added the following passage to clarify data saturation: “The sample size was determined by data saturation: we continued to interview health professionals across categories until no new themes or insights emerged from subsequent interviews.”
- Policy implications: In the discussion, we have extended the implications for policy and what can be done by Ministries of Health and hospital administrators.
- Potential for the POCT in the health system: Thank you for this recommendation. We

have added this to the discussion.

- Reflexivity: we have added more detail on author reflexivity in the section on data generation and on data analysis and interpretation.

Reviewer: 3
Dr. Miroslav Barták, Charles University and General University Hospital in Prague
Comments to the Author:
Thank you for the opportunity to write this review and to the authors for covering this topic. The article certainly provides a new and important perspective on the topic of methanol poisoning, and the interviews conducted are highly informative. On the other hand, I see some fundamental problems. The first is the question of literature review. The literature on methanol poisoning is quite extensive, but the introductory section of the article fails to mention a number of publications that would be worth citing, and there is no mention of the research strategy. The design of the study is appropriate for the purpose of the article, but I believe that some of the information provided in CONSEQ is missing from the paper or is described very briefly. The methodological approach to the analysis itself is described in very general terms; the appendix contains an illustrative list of questions, but not the entire coding framework. It is not entirely clear how many people coded and what the specific analysis process was. The processing of the results should be much clearer, and visualization could make this part of the text more readable. I find the discussion insufficient, and the definition of strengths and weaknesses is inadequate. The conclusions are rather general and do not fully correspond to the possibilities of qualitative analysis, and they are very short.
With better processing of the qualitative analysis, I consider the survey to be beneficial, but a complete revision of the text is necessary.

Thank you for reviewing our manuscript and for your feedback. These insights have supported us to revise and improve the manuscript. Based on this, we have made the following revisions:

- Introduction: We have added the research strategy in the introduction.
- Introduction literature review: Thank you for this observation. We were previously trying to be judicious in citing work, but we have now made this more comprehensive.
- COREQ checklist: We have added further information in the methods, including:
    - Researcher genders: the team of researchers primarily involved in data collection was composed of one female and one male. We have added this.
    - Participant knowledge of interviewers and interviewer characteristics: We have added the following to the methods: “We approached interview participants with a participant information sheet and explained the study's objectives, potential uses of the results, and the voluntary nature of participation. We also explained our reasons for our interest in the topic, how it was embedded in the trial of a new diagnostic device, and our (the researchers’) basic background details.”
    - Approaching participants: We approached potential participants face-to-face. We have added this to the methods.
    - Location of interview and presence of others: We have added the following: “Interviews were carried out in a private location at the participant’s workplace.”
- Data analysis: We have revised the text in the methods section to describe the data analysis process in greater detail. For this paper, we have decided to present illustrative quotes for the main themes and sub-themes in a table included in the main text of the article. We therefore did not include a coding tree, as this would be repetitive. In addition to this table, we are also providing the full interview guidelines as supplementary files.
- Discussion: We have made significant revisions to the discussion section and explored the themes in more detail.

- Strengths and limitations: We have expanded and provided more details of the strengths and limitations in the discussion section.
- Conclusion: We have revised and expanded the conclusion section as suggested.

Reviewer: 1
If you have selected 'Yes' above, please provide details of any competing interests.: Not applicable

Reviewer: 2
If you have selected 'Yes' above, please provide details of any competing interests.: Not applicable

Reviewer: 3
If you have selected 'Yes' above, please provide details of any competing interests.: ´Not applicable'

VERSION 2 - REVIEW

**Reviewer**	**3**
**Name**	**Barták, Miroslav**
**Affiliation**	**Charles University and General University Hospital in Prague, Department of Addictology**
**Date**	**18-Mar-2026**
**COI**	

Thank you for the opportunity to review the revised version of the article. The authors have incorporated my comments into the text and have also provided a sufficient explanation of the changes that were made. Based on these revisions, I recommend the article for publication.
